# Allele-specific multi-sample copy number segmentation in ASCAT

**DOI:** 10.1093/bioinformatics/btaa538

**Published:** 2021-05-25

**Authors:** Edith M. Ross, Kerstin Haase, Peter Van Loo, Florian Markowetz

**Affiliations:** Cancer Research UK Cambridge Institute, University of Cambridge, Cambridge CB2 0RE, UK; The Francis Crick Institute, London NW1 1AT, UK; The Francis Crick Institute, London NW1 1AT, UK; Cancer Research UK Cambridge Institute, University of Cambridge, Cambridge CB2 0RE, UK

## Abstract

**Motivation:**

Allele-specific copy number alterations are commonly used to trace the evolution of tumours. A key step of the analysis is to segment genomic data into regions of constant copy number. For precise phylogenetic inference, breakpoints shared between samples need to be aligned to each other.

**Results:**

Here, we present asmultipcf, an algorithm for allele-specific segmentation of multiple samples that infers private and shared segment boundaries of phylogenetically related samples. The output of this algorithm can directly be used for allele-specific copy number calling using ASCAT.

**Availability and implementation:**

asmultipcf is available as part of the ASCAT R package (version ≥2.5) from github.com/Crick-CancerGenomics/ascat/.

## 1 Introduction

Allele-specific copy number alterations (CNAs) are commonly used to trace the evolution of tumours. One of the most frequently used algorithms to infer these copy number changes is ASCAT (Van Loo *et al.*, 2010), which segments each sample separately. Due to measurement noise, the inferred locations of breakpoints shared between samples often differ. These differences can impair analyses of phylogenetic relationships between the samples, because evolutionary methods depend on the assumption that shared breakpoints appear at exactly the same location. Previous approaches to address this problem include extensive experimental breakpoint validation (Schwarz *et al.*, 2015), an expensive approach that is not always feasible, or size-based heuristic filters (Mangiola *et al.*, 2016). Another approach infers allele and clone-specific CNA from multi-sample data by binning without segmentation (Zaccaria and Raphael, 2018).

To rigorously address the problem of multi-sample breakpoint detection, we have developed asmultipcf (allele-specific multi-sample piecewise constant fitting), a robust allele-specific multi-sample segmentation algorithm that is tightly integrated into the ASCAT framework (Van Loo *et al.*, 2010). The ability of asmultipcf to improve phylogenetic inference was shown in a large case study on 181 samples from 10 patients with lethal metastatic breast cancer (De Mattos-Arruda *et al.*, 2019).

## 2 Approach

asmultipcf incorporates and extends two copy number segmentation algorithms previously developed by Nilsen *et al.* (2012), which leverage vector operations for efficient implementation: first, aspcf (an allele-specific segmentation method for single samples), and second, multipcf (a multi-sample segmentation method, which is not allele-specific). Additionally, asmultipcf handles missing values, making extensive data filtering unnecessary.

### 2.1 Input data

For each sample, the following input data are required across germline heterozygous sites: (i) log ratios (logR), representing log-transformed copy numbers derived from sequencing depth or single nucleotide polymorphism (SNP) array data, and (ii) B allele frequencies (BAF), describing the allelic imbalance of SNPs. The algorithm presented here can handle missing values and thus loci with incomplete data across samples do not need to be excluded.

### 2.2 Pre-processing

asmultipcf uses the same pre-processing steps as the allele-specific single sample algorithm of Nilsen *et al.* (2012), including (i) mirroring BAFs to obtain a single track in regions of allelic imbalance and (ii) removing extreme outliers from logR and BAF data [see Nilsen *et al.* (2012) for details]. Given *n* samples across *p* SNP loci, the pre-processing yields a single matrix $Y=(y_{ij})\in R^{2n\times p}$ that contains both logR and BAF values.

### 2.3 An exact algorithm for weighted segmentation

We evaluate the fit of a segmentation solution to the data with a weighted least squares function that models missing values in the data matrix. A weight matrix $W=(w_{ij})\in R^{2n\times p}$ is derived by assigning *w_ij_* a weight of 0 if *y_ij_* is missing and 1 otherwise. Then all missing values in **Y** are assigned an arbitrary [non-not assigned (NA)] value. Our aim is to find a segmentation $S=\{I_{1},\ldots ,I_{M}\}$ that minimizes the cost function

(1) $$L(S|Y,W,\gamma )=\sum_{i=1}^{2n}L(S|y_{i.},w_{i.},\gamma )$$

 

(2) $$=\sum_{i=1}^{2n}\sum_{I\in S}\sum_{j\in I}w_{ij}{\left(y_{ij}-{\bar{y}}_{i,I}\right)}^{2}+\gamma |S|,$$

where the best fit on a given segment *I* is the weighted average of the observations on that segment

$${\bar{y}}_{i,I}=\sum_{j\in I}w_{ij}y_{ij}/\sum_{j\in I}w_{ij}$$

and where *γ* is a penalty parameter that controls the number of segments. Expanding the square in (2) and omitting the term independent of *S*:

$$L\prime (S|Y,W,\gamma )=-\sum_{i=1}^{2n}\sum_{I\in S}\frac{{\left(\sum_{j\in I}w_{ij}y_{ij}\right)}^{2}}{\sum_{j\in I}w_{ij}}+\gamma |S|.$$

To find an optimal solution to the cost function, we adapt the dynamic programming algorithm of Nilsen *et al.* (2012) to our weighted problem. The algorithm iteratively minimizes the total errors $e_{k}$ at locus *k* across all samples using the errors $e_{k-1}$ up to *k*, the costs of the current segments, $d_{k}$, and the penalty *γ*, together with intermediate variables $A_{k}$ and $C_{k}$:

**Algorithm 1: asmultipcf**

*Input:* Matrix **Y** of log-transformed copy numbers and B allele frequencies; weight matrix **W**; penalty γ>0;

*Output:* Segment start indices and segment averages

1. Initialize $A_{0}=[\;],\;C_{0}=[\;],\;e_{0}=0$ and iterate for k=1,…,p
  -
$A_{k}=[A_{k-1}0]+w_{.k}y_{.k}$

  -
$C_{k}=[C_{k-1}0]+w_{.k}$

  -
$d_{k}=-1^{T}\left(A_{k}{}^{\circ}A_{k}{}^{\circ}C_{k}^{{}^{\circ}-1}\right)$ where ° denotes an element- wise matrix product and $C_{k}^{{}^{\circ}-1}$ the element-wise inverse
  -
$e_{k}=\left[e_{k-1}\text{min}(d_{k}+e_{k-1}+\gamma )\right]$ storing also the index $t_{k}\in 1,\ldots ,k$ at which the minimum in the last step is achieved.
2. Find segment start indices from right to left as $s_{1}=t_{p},\;s_{2}=t_{s_{1}-1},\;\ldots$, *s_M_*=1, where M≤1.
3. Find segment averages
  $${\bar{y}}_{m}=\frac{\left(w_{.s_{m}}y_{.s_{m}}+\cdots +w_{.s_{m-1}-1}y_{.s_{m-1}-1}\right)}{\left(w_{.s_{m}}+\cdots +w_{.s_{m-1}-1}\right)}.$$

### 2.4 A heuristic algorithm for large data sets

Algorithm 1 is of order $O(p^{2})$, which means that the segmentation becomes computationally expensive for long sequences. However, instead of allowing breakpoints at any of the *p* positions, we can pre-select potential breakpoints and thereby reduce the runtime to $O(q^{2})$ where *q* is the number of potential breakpoints. To identify potential breakpoints, different heuristics can be used. Here, we apply Algorithm 1 to overlapping subsequences (length 5000 with an overlap of 1000), combine all of the inferred breakpoints and use them as input for the subsequent global segmentation. Algorithm 2 describes the fast heuristic version of asmultipcf.

**Algorithm 2: Fast asmultipcf**

*Input:* Matrix **Y** of log-transformed copy numbers and B allele frequencies; weight matrix **W**; penalty γ>0;

*Output:* Segment start indices and segment averages

1. Split data set into overlapping subsequences and apply steps 1 and 2 of Algorithm 1 to each of them in order to find potential breakpoints *r*_0_, *r*_1_, …, *r_q_* where $r_{0}=1$ and $r_{1}=p+1$.
2. Aggregate sequences between breakpoints by setting $x_{ik}=\sum_{j=r_{k-1}}^{r_{k}-1}w_{ij}y_{ij}$ and $v_{ik}=\sum_{j=r_{k-1}}^{r_{k}-1}w_{ij}$.
3. Calculate segmentation solution by using the aggregated matrices **X** and $V\in R^{2n\times q}$ as input to Algorithm 1 instead of **Y** and **W**, respectively.

### 2.5 Post-processing

Both algorithms yield a single segmentation solution *S* for all samples. However, we expect that only some of the segments will be shared between all samples while others will be private. While ASCAT can be run directly on the global segmentation solution, removing unnecessary breakpoints on a per-sample base can reduce noise in the segment average estimates by generating larger segments. To refine breakpoints individually for each sample, we simply use the breakpoints inferred from the multi-sample segmentation and rerun steps 2 and 3 of Algorithm 2 on each sample individually based on these potential breakpoints.

### 2.6 Implementation

asmultipcf is part of the ASCAT R package from version 2.5 onwards. The asmultipcf function contains a parameter to select whether the exact or the fast algorithm should be run, as well as an option to include the per-sample breakpoint refinement. Furthermore, samples can be weight adjusted to account for quality differences in the data. The manual contains example use cases, including a comparison to HATCHet (Zaccaria and Raphael, 2018).

## 3 Discussion

The independent segmentation of related samples can artificially inflate tumour heterogeneity. The algorithm presented here addresses this problem by joint segmentation. While this approach can potentially underestimate tumour heterogeneity, because CNAs that are shared by many samples are more likely to be detected than CNAs that are private or shared by only few samples, in practice, the penalty parameter *γ* can be adjusted to ensure sensitivity. Overall, asmultipcf substantially improves the analysis of copy number changes of multiple samples.

## Funding

This research was supported by the Cancer Research UK Cambridge Institute with core grant C14303/A17197 and the Francis Crick Institute with core funding from Cancer Research UK [FC001202], the UK Medical Research Council [FC001202] and the Wellcome Trust [FC001202]. P.V.L. is a Winton Group Leader, F.M. is a Royal Society Wolfson Research Merit award holder.

*Conflict of Interest*: none declared.
